# Supplementary material for: Inflammatory factors and risk of lung adenocarcinoma: a Mendelian randomization study mediated by blood metabolites
Source: Front Endocrinol (Lausanne). 2024 Aug 27;15:1446863. doi: 10.3389/fendo.2024.1446863 (PMC11384989; doi:10.3389/fendo.2024.1446863)
Supplement: Supplementary file 1 [file DataSheet1.zip › supplementary files/S2/GCST90201000/sensitivity-analysis.pdf]

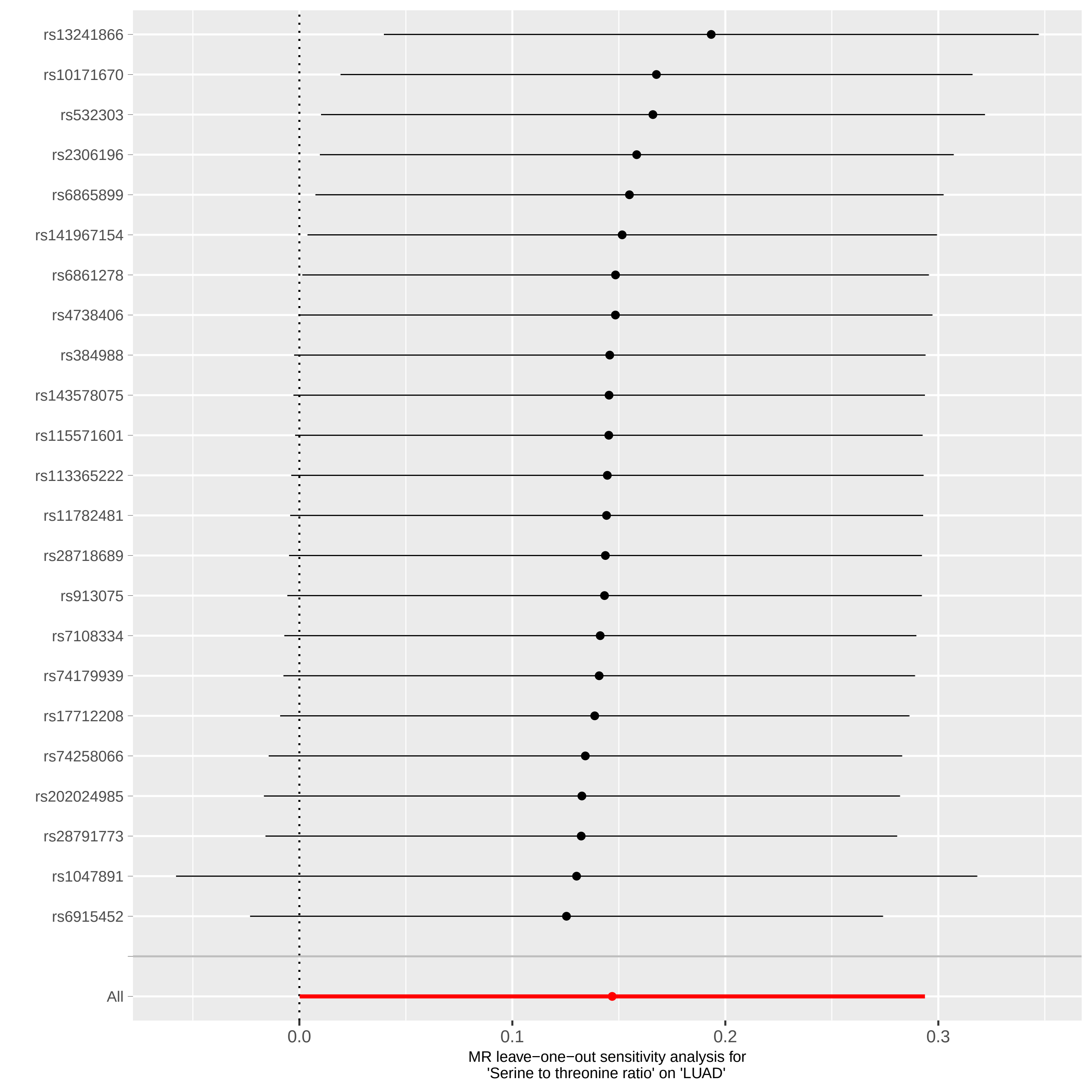
rs13241866
rs10171670
rs532303
rs2306196
rs6865899
rs141967154
rs6861278
rs4738406
rs384988
rs143578075
rs115571601
rs113365222
rs11782481
rs28718689
rs913075
rs7108334
rs74179939
rs17712208
rs74258066
rs202024985
rs28791773
rs1047891
rs6915452
All
0.0
0.1
0.2
0.3
MR leave−one−out sensitivity analysis for
'Serine to threonine ratio' on 'LUAD'
